# Solubility of Omeprazole Sulfide in Different Solvents at the Range of 280.35–319.65 K

**DOI:** 10.1007/s10953-013-0110-y

**Published:** 2013-11-12

**Authors:** Yihua Li, Wenge Yang, Tuan Zhang, Chaoyuan Wang, Kai Wang, Yonghong Hu

**Affiliations:** College of Biotechnology and Pharmaceutieal Engineering, Nanjing University of Technology, No. 200, North Zhongshan Road, Nanjing, 210009 China

**Keywords:** Omeprazole sulfide, Solubility, Gravimetric method, Purification, Solution thermodynamics

## Abstract

Solubility data were measured for omeprazole sulfide in ethanol, 95 mass-% ethanol, ethyl acetate, isopropanol, methanol, acetone, *n*-butanol and *n*-propanol in the temperature range from 280.35 to 319.65 K by employing the gravimetric method. The solubilities increase with temperature and they are in good agreement with the calculated solubility of the modified Apelblat equation and the *λh* equation. The experimental solubility and correlation equation in this work can be used as essential data and model in the purification process of omeprazole sulfide. The thermodynamic properties of the solution process, including the Gibbs energy, enthalpy, and entropy were calculated using the van’t Hoff equation.

## Introduction

**Fig. 1:**
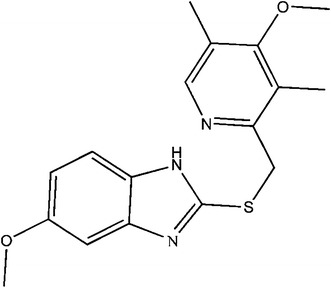
Chemical structure of omeprazole sulfide

Omeprazole sulfide, an amorphous colorless or white powder, is odorless and stable in air. Omeprazole sulfide (C_17_H_19_N_3_O_2_S, FW 329.42, CAS Registry No. 73590-85-9, structure shown in Fig. 1) is a degradation product of omeprazole. It has been reported to be an antagonist for AHR in HepG2 cells [1] and it acts as an agonist for AHR in human hepatocytes [2]. AHR is aryl hydrocarbon receptor, a mediated transactivation receptor-type transcription factor. Omeprazole sulfide is also an important intermediate in pharmaceuticals. It is usually used to synthesize omeprazole and esomeprazole, which are used in the treatment of gastric acid related disorders [3–5] and are effective in the control of gastric acidity of patients with Zollinger–Ellison syndrome, as well as in patients that do not respond well to histamine H_2_ receptor antagonists [3, 6]. In addition, gastrointestinal (GI) diseases account for substantial morbidity, mortality, and cost [7], which leads to the increased demand for the related drugs, such as omeprazole capsules, omeprazole enteric-coated tablets, esomeprazole sodium for injection, and esomeprazole magnesium enteric-coated tablets. It results in great demand for this key intermediate.

Omeprazole sulfide is synthesized using 2-mercapto-5-methoxybenzene imidazole and 2-chloromethyl-4-methoxy-3,5-lutidine [8] or 4-methoxy-3,5-dimethyl-2-pyridine-methyl bromide [9] as substrate. It should be purified by dissolution, crystallization and separation. Crystallization processes are the critical steps that determine the quality of the product [10] of omeprazole sulfide to provide sufficient purity for the next reaction. So it is very important to know the solubility of omeprazole sulfide as a function of temperature and solvent composition in selected solvents required for the preparation and purification of the products [11]. Moreover, no literature study has reported the correlation between the solubility and temperature or the solvent composition. The most basic information for solving the solvent selection problem is the basic physical properties and solubility data [12]. Therefore we measured and correlated the solubility data of omeprazole sulfide in different solvents (ethanol, 95 mass-% ethanol, ethyl acetate, isopropanol, methanol, acetone, *n*-butanol and *n*-propanol) over the temperature range 280.35–319.65 K under atmospheric pressure by the gravimetric method [13, 14]. Thus, systematic and necessary information on the crystallization of omeprazole sulfide was obtained. For predicting the solubility of a solute in different solvents, several methods have been presented [11, 15]. This study used the modified Apelblat equation and the Buchowski–Ksiazaczak *λh* equation to correlate and predict the solubility of omeprazole sulfide in different solvents.

## Experimental

### Materials

A white crystalline powder of omeprazole sulfide was supplied by Shanghai Lingfeng Chemical Reagent Co., China. The mass fraction was higher than 0.995, measured by high performance liquid chromatography (HPLC type DIONEX P680 DIONEX Technologies). The melting temperature was 392.15 K determined by differential scanning calorimeter (Netzsch DSC 204). The ethanol, ethyl acetate, isopropanol, methanol, acetone, *n*-butanol and *n*-propanol used for experiment were all analytical purity grade with mass fraction purity higher than 0.995 except for 95 % ethanol. They were supplied by Shanghai Shenbo Chemical Co., Ltd. and used without further purification.

### Methods

The solubility of omeprazole sulfide was determined by a gravimetric method. The balance used in these experiments was an analytical balance with an uncertainty of ±0.0001 g (Sartorius, BS210S). 8 mL of solvent and a rotor were put into a 10 mL glass test tube with stopper, and then excess omeprazole sulfide was added into the glass test tube [16]. The test tubes were partly immersed in a constant-temperature bath. The temperature was controlled by a jacketed vessel with water circulated through the outer jacket from a super thermostatic water-circulator bath (type DC-2006 Ningbo XinYi Biotechnology Co., Ltd.). Meanwhile, the inner chamber of the vessel needs a mercury-in-glass thermometer with an uncertainty of ±0.05 K (calibrated by using a standard thermometer) for measuring the solution equilibrium temperature. Continuous stirring was adopted for fully mixing the suspension with a magnetic stirrer at each temperature [17].

In order to make sure that the solution system established the solid–liquid equilibrium, a stirring time of 12 h was provided, and then the solution was kept still about 3 h to ensure a dynamic balance was established between the dissolution and the crystallization processes. Then, about 1 mL of supernatant was taken from the test tube and transferred to a previously weighed 5 mL sampling vial using a pre-warmed pipette [17, 18] carefully and quickly. Subsequently, the mass of the sample was determined by weighing the sampling vial again. Then the sampling vial was put into a dryer to dry at room temperature. Afterwards the sampling vial was weighed on a regular basis until it reached a constant weight. Each experiment was repeated at least twice to check the repeatability of the solubility determination, and three samples were taken for each solvent at each temperature [19] and the mean value was considered as the solubility. The saturated mole fraction solubility (*x*) is obtained from the following equation:

1 $$ x = \frac{{{{m_{1} } \mathord{\left/ {\vphantom {{m_{1} } {M_{1} }}} \right. \kern-0pt} {M_{1} }}}}{{{{m_{1} } \mathord{\left/ {\vphantom {{m_{1} } {M_{1} }}} \right. \kern-0pt} {M_{1} }} + {{m_{2} } \mathord{\left/ {\vphantom {{m_{2} } {M_{2} }}} \right. \kern-0pt} {M_{2} }}}} $$

where *m*_1_ represents the mass of solute and *m*_2_ the mass of solvents. *M*_1_ is the molecular mass of solute and *M*_2_ that of the solvent.

## Results and Discussion

### Solubility Data and Correlation Models

The saturated mole fraction solubility (*x*) and the calculated solubility values (*x* ^c^) of omeprazole sulfide in ethanol, 95 % ethanol, ethyl acetate, isopropanol, methanol, acetone, *n*-butanol and *n*-propanol in the temperature range from 280.35 to 319.65 K are presented in Table 1.

**Table 1:** The saturated mole fraction solubility (*x*) and the calculated solubility values (*x* ^c^) by Eqs. 2 and 3 of omeprazole sulfide, in different solvents at the temperature range from 280.35 to 319.65 K

*T*/K	*x*	Equation 2		Equation 3	
		*x* ^c^	100*RD*	*x* ^c^	100*RD*
Ethanol					
280.35	0.0144	0.0148	−2.438	0.0142	1.507
284.35	0.0189	0.0193	−2.103	0.0189	0.264
288.15	0.0248	0.0247	0.415	0.0244	1.615
292.25	0.0323	0.0319	1.145	0.0318	1.490
296.65	0.0412	0.0417	−1.199	0.0418	−1.417
300.25	0.0511	0.0514	−0.734	0.0517	−1.183
303.15	0.0606	0.0606	−0.164	0.0610	−0.670
307.65	0.0775	0.0776	−0.145	0.0780	−0.569
311.15	0.0946	0.0934	1.269	0.0937	1.018
315.15	0.1151	0.1145	0.576	0.1145	0.571
319.65	0.1415	0.1424	−0.685	0.1420	−0.398
95 mass-% Ethanol					
280.35	0.0101	0.0103	−1.912	0.0100	0.680
284.35	0.0137	0.0138	−0.882	0.0136	0.570
288.15	0.0178	0.0181	−1.881	0.0180	−1.194
292.25	0.0240	0.0240	−0.307	0.0240	−0.180
296.65	0.0325	0.0323	0.861	0.0323	0.673
300.25	0.0409	0.0407	0.503	0.0408	0.224
303.15	0.0489	0.0489	−0.043	0.0490	−0.315
307.65	0.0641	0.0643	−0.213	0.0644	−0.379
311.15	0.0791	0.0789	0.309	0.0789	0.259
315.15	0.0989	0.0989	−0.002	0.0988	0.053
319.65	0.1258	0.1260	−0.101	0.1259	−0.037
Ethyl acetate					
280.35	0.0033	0.0032	2.293	0.0033	−0.852
284.35	0.0040	0.0040	2.238	0.0040	0.389
288.15	0.0049	0.0048	1.478	0.0048	0.561
292.25	0.0058	0.0059	−1.126	0.0059	−1.314
296.65	0.0073	0.0073	0.522	0.0072	0.839
300.25	0.0085	0.0086	−1.649	0.0086	−1.107
303.15	0.0098	0.0099	−0.273	0.0098	0.321
307.65	0.0122	0.0121	0.723	0.0120	1.233
311.15	0.0142	0.0142	0.586	0.0141	0.915
315.15	0.0165	0.0169	−2.217	0.0169	−2.192
319.65	0.0208	0.0206	1.074	0.0207	0.669
Isopropanol					
280.35	0.0032	0.0033	−2.520	0.0031	2.624
284.35	0.0044	0.0045	−2.152	0.0043	1.309
288.15	0.0058	0.0059	−1.943	0.0058	0.218
292.25	0.0079	0.0080	−1.311	0.0079	−0.251
296.65	0.0108	0.0110	−1.907	0.0109	−1.683
300.25	0.0140	0.0141	−0.475	0.0141	−0.698
303.15	0.0172	0.0171	0.870	0.0172	0.439
307.65	0.0234	0.0230	1.764	0.0231	1.240
311.15	0.0286	0.0287	−0.405	0.0289	−0.842
315.15	0.0370	0.0369	0.363	0.0369	0.204
319.65	0.0482	0.0484	−0.361	0.0482	−0.039
Methanol					
280.35	0.0127	0.0135	−5.842	0.0125	2.056
284.35	0.0178	0.0183	−2.814	0.0175	1.973
288.15	0.0239	0.0242	−1.458	0.0236	1.161
292.25	0.0322	0.0325	−0.975	0.0322	−0.012
296.65	0.0442	0.0440	0.444	0.0441	0.293
300.25	0.0559	0.0558	0.078	0.0562	−0.550
303.15	0.0668	0.0673	−0.629	0.0678	−1.411
307.65	0.0887	0.0888	−0.080	0.0894	−0.768
311.15	0.1103	0.1091	1.052	0.1096	0.627
315.15	0.1380	0.1367	0.974	0.1367	0.940
319.65	0.1721	0.1735	−0.862	0.1728	−0.446
Acetone					
280.35	0.0050	0.0050	0.325	0.0051	−0.425
284.35	0.0065	0.0065	0.257	0.0066	−0.238
288.15	0.0083	0.0083	0.405	0.0083	0.108
292.25	0.0108	0.0107	0.908	0.0107	0.779
296.65	0.0140	0.0140	0.339	0.0140	0.337
300.25	0.0172	0.0172	−0.102	0.0172	−0.035
303.15	0.0203	0.0204	−1.543	0.0203	−1.444
307.65	0.0263	0.0262	0.335	0.0262	0.445
311.15	0.0319	0.0317	0.746	0.0317	0.832
315.15	0.0390	0.0392	−0.639	0.0392	−0.611
319.65	0.0496	0.0495	0.181	0.0495	0.103
*n-*Butanol					
280.35	0.0103	0.0100	2.607	0.0102	0.882
284.35	0.0134	0.0131	1.640	0.0133	0.337
288.15	0.0171	0.0169	0.958	0.0171	0.045
292.25	0.0218	0.0220	−0.809	0.0221	−1.334
296.65	0.0292	0.0289	1.078	0.0289	0.928
300.25	0.0356	0.0359	−0.641	0.0358	−0.550
303.15	0.0425	0.0425	−0.140	0.0424	0.094
307.65	0.0549	0.0549	−0.054	0.0547	0.291
311.15	0.0661	0.0666	−0.655	0.0663	−0.334
315.15	0.0824	0.0823	0.061	0.0822	0.204
319.65	0.1039	0.1037	0.225	0.1039	−0.052
*n-*Propanol					
280.35	0.0076	0.0077	−1.845	0.0076	−0.541
284.35	0.0101	0.0103	−1.479	0.0102	−0.767
288.15	0.0134	0.0134	−0.416	0.0134	−0.106
292.25	0.0178	0.0177	0.657	0.0177	0.690
296.65	0.0234	0.0236	−0.837	0.0236	−0.954
300.25	0.0296	0.0297	−0.158	0.0297	−0.310
303.15	0.0359	0.0355	1.109	0.0356	0.972
307.65	0.0470	0.0466	0.827	0.0466	0.755
311.15	0.0567	0.0571	−0.600	0.0571	−0.611
315.15	0.0713	0.0715	−0.361	0.0715	−0.323
319.65	0.0914	0.0913	0.154	0.0913	0.173

The relationship between temperature and mole fraction solubility in different solvents is described by the modified Apelblat equation, which is a semiempirical equation derived from the Clausius–Clapeyron equation [20, 21], which is as follows:

2 $$ \ln \, x = A + \frac{B}{T} + C\ln T $$

where *T* represents the absolute temperature, *A, B* and *C* are the model parameters, and *x* is the mole fraction solubility of omeprazole sulfide. The constants *A* and *B* represent the variation in the solution activity coefficient and provide an indication of the effect of non-ideal solution behavior on the solute solubility, while the constant *C* reflects the temperature influence on the enthalpy of fusion [22]. The adjustable parameters *A, B* and *C* can be obtained by fitting the experimental solubility data.

The Buchowski–Ksiazaczak *λh* equation is an alternate way to describe solid–liquid equilibrium behavior of omeprazole sulfide, as first proposed by Buchowski et al. [23]. The experimental data for many systems can be well represented by the Buchowski–Ksiazaczak *λh* equation with only two parameters *λ* and *h* [24–27]. In this paper, the solubility data were also correlated with the Buchowski–Ksiazaczak *λh* equation:

3 $$ \ln \left[ {1 + \frac{\lambda (1 - x)}{x}} \right] = \lambda h\left[ {\frac{1}{T} - \frac{1}{{T_{m} }}} \right] $$

where *T* represents the system temperature, *T*_*m*_ is the melting point temperature of omeprazole sulfide in Kelvin, *x* is the mole fraction solubility of omeprazole sulfide and *λ* and *h* are the model parameters determined by the experimental data in the system.

Using the values in Table 1, the parameters of *A, B* and *C* were estimated and presented in Table 2, and the parameters of *λ* and *h* are listed in Table 3, together with the root-mean-square deviations (RMSDs) and the relative average deviations (RADs).

**Table 2:** Parameters of Eq. 2 for mole fraction solubility of omeprazole sulfide in various solvents

Solvent	*A*	*B*	*C*	10^2^ RMSD	10^2^ RAD
Ethanol	169.713	−12176.733	−23.160	1.082	0.973
95 % Ethanol	100.445	−9611.497	−12.564	0.521	0.408
Ethyl acetate	−105.237	932.180	17.069	1.069	0.945
Isopropanol	121.765	−10949.042	−15.700	1.149	0.868
Methanol	290.941	−18217.582	−40.870	1.125	0.930
Acetone	−0.480	−4587.603	2.051	0.627	0.487
*n-*Butanol	31.893	−6080.526	−2.624	0.610	0.459
*n-*Propanol	86.751	−8848.671	−10.657	0.632	0.564

**Table 3:** Parameters of Eq. 3 for mole fraction solubility of omeprazole sulfide in various solvents

Solvent	*λ*	*h*	10^2^ RMSD	10^2^ RAD
Ethanol	3.582	1504.278	1.217	0.988
95 % Ethanol	4.097	1447.915	0.918	0.639
Ethyl acetate	0.202	20201.853	1.469	1.289
Isopropanol	1.770	3496.429	1.496	1.279
Methanol	7.224	853.657	2.100	1.382
Acetone	1.012	5153.445	0.659	0.525
*n-*Butanol	2.614	2091.202	1.093	0.806
*n-*Propanol	2.703	2129.557	0.922	0.767

The RMSD is defined as follows:

4 $$ {\text{RMSD}} = \sqrt {\frac{{\sum\nolimits_{i = 1}^{N}
{(x^{\text{e}} - x^{\text{c}} )^{2} } }}{N}} $$

The RAD is defined as follows:

5 $$ {\text{RAD}} = \frac{1}{N}\sum\limits_{i = 1}^{N} {\left|
{\frac{{x^{\text{e}} - x^{\text{c}} }}{{x^{\text{e}} }}} \right|}
$$

where *N* is the number of experimental points obtained in each set, which equals the number of temperatures used, *x* ^c^ represents the calculated solubility values and *x* ^e^ the experimental solubilities.

The relative deviations (RDs) between the experimental values and the calculated values are also presented in Table 1. The RDs are given as:

6 $$ {\text{RD}} = \frac{{x^{\text{e}} - x^{\text{c}}
}}{{x^{\text{e}} }} $$

where *x* ^c^ represents the calculated solubilities and *x* ^e^ the experimental values.

**Fig. 2:**
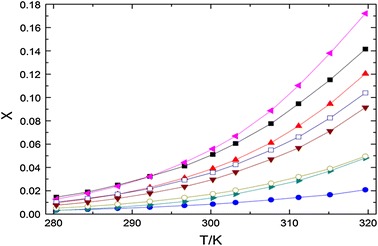
Solubilities of omeprazole sulfide in different solvents at atmospheric pressure. *Left pointing filled triangle* methanol, *filled square* ethanol, *filled triangle* 95 % ethanol, *open square* *n-*butanol, *inverted triangle* *n-*propanol, *open circle* acetone, *right pointing filled triangle* isopropanol, *filled circle* ethyl acetate. *Solid lines* calculated using Eq. 2

The *x*/*T* curves of omeprazole sulfide, measured in all the solvents studied, are presented in Fig. 2. As we can see from Fig. 2, all the solubility curves are similar, with low solubilities at low temperature, which increase at higher temperatures [28]. The solubility is a function of temperature and increases with increasing temperature. From Fig. 2, it can be seen that the solubility of omeprazole sulfide is relatively low in acetone, isopropanol and ethyl acetate at all temperatures. The solubility in ethyl acetate has the smallest percentage change, while the solubility in acetone is substantially the same as in isopropanol. As for the other five solvents, the solubilities are more sensitive to temperature, especially methanol in which the solubility varies much more obviously with temperature, ethanol and 95 % ethanol. So methanol, ethanol and 95 % ethanol can be used to recrystallize omeprazole sulfide. However, in industrial production, taking the safety and cost into account, ethanol or 95 % ethanol presents a potential advantage in the crystallization process of omeprazole sulfide.

As we all know, methanol, ethanol, propanol and butanol are protic solvents that have a hydrogen atom bound to an oxygen (in a hydroxyl group). The molecules of such solvents can readily donate protons and interact with solute molecules by hydrogen bonding. The hydrogen bonds could increase the solubility of the solute. This may be the reason that the solubility is relatively high in methanol, ethanol, 95 % ethanol, *n*-butanol and *n*-propanol. The solubility in methanol and ethanol is higher than that in *n*-propanol and *n*-butanol. This phenomenon can be explained by the fact that when the alcohol chain length increases, the hydrogen bonds with alcohols are weakened [29]. However, the solubility in isopropanol is relatively low, perhaps because the hydroxy of the isopropanol molecules is located between two CH_3_ groups, which hinder the interaction of the H and N atoms. The solubility of omeprazole sulfide is lower in 95 % ethanol than in ethanol. The reason may be that the sulfide compounds are practically insoluble in water.

As can be seen from Tables 1, 2 and 3, the calculated data of omeprazole sulfide in a total of eight solvents show good agreement with the experimental data. For the modified Apelblat equation, as we can see from Tables 1 and 2, the RADs are 0.97, 0.41, 0.95, 0.87, 0.93, 0.49, 0.46 and 0.56 %, respectively and the absolute values of RDs do not exceed 2.6 %, which indicates that all the solubility data can be calculated in the selected solvents when the modified Apelblat equation is used. The same is true for analyzing the solubility data and the parameters that fitted the *λh* equation. Furthermore, from Tables 2 and 3, all the solubilities are calculated with reasonable RMSD and the average RMSDs are 0.85 and 1.2, for the modified Apelblat and Buchwski–Ksiazaczak *λh* equations respectively. Therefore the regression result of the modified Apelblat equation is more accurate than the Buchowski–Ksiazaczak *λh* equation. Compared to the Buchowski–Ksiazaczak *λh* equation, the modified Apelblat equation is proposed for solid–liquid equilibria, and it is widely accepted as being capable of dealing with solvent systems. Therefore, the measured solubility data and the correlation equation in this work can be applied to the design and optimization for the extraction and purification process of omeprazole sulfide [19].

### Thermodynamic Properties for the Solution

The temperature dependence of the solubility allows a thermodynamic analysis that permits insight into the molecular mechanisms involved in the solution processes [30]. In this study, the thermodynamic functions in the process of solution of omeprazole sulfide are calculated on the basis of the solubility of omeprazole sulfide in different solvents. The standard molar enthalpy of solution $\left( {\Updelta H_{\text{soln}}^{\text{o}} } \right)$ is accessible from this equation, which is the van’t Hoff analysis and defined as [30–32]:

7 $$ \Updelta H_{\text{soln}}^{\text{o}} = - R\left( {\frac{{\partial \ln x_{1} }}{{\partial \left( {{1 \mathord{\left/ {\vphantom {1 T}} \right. \kern-0pt} T}} \right)}}} \right) $$

where *x*_1_ is the mole fraction solubility, *R* represents the universal gas constant (8.314 J·K^−1^·mol^−1^) and *T* is the absolute temperature. The standard molar enthalpy change of solution, $\Updelta H_{\text{soln}}^{\text{o}}$, is generally obtained from the slope of the solubility curve in a so-called van’t Hoff plot where ln *x* is plotted against *T* ^−1^. Over a limited temperature interval, the heat capacity change of a solution may be assumed to be constant, hence the derived values of $\Updelta H_{\text{soln}}^{\text{o}}$ will also be valid for the mean temperature, *T*_mean_ = 300 K [33]. Equation 7 can also be written as:

8 $$ \Updelta H_{\text{soln}}^{\text{o}} = - R\left( {\frac{\partial \ln x}{{\partial \left( {{1 \mathord{\left/ {\vphantom {1 T}} \right. \kern-0pt} T} - {1 \mathord{\left/ {\vphantom {1 {T_{\text{mean}} }}} \right. \kern-0pt} {T_{\text{mean}} }}} \right)}}} \right) $$

**Fig. 3:**
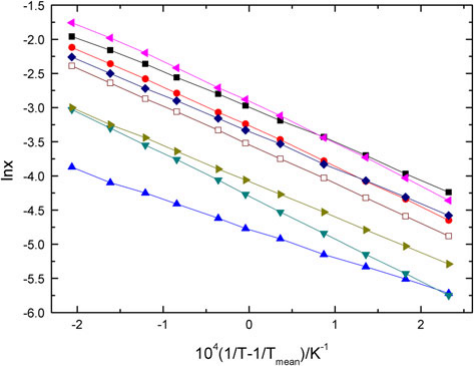
Mole fraction solubility (ln *x*) of omeprazole sulfide in different solvents against 10^4^ (1/*T* − 1/*T*_mean_) with a straight line to correlate the data. *Left pointing filled triangle* methanol, *filled square* ethanol, *filled circle* 95 % ethanol, *diamond* *n-*butanol, *open square* *n-*propanol, *right pointing filled triangle* acetone, *inverted triangle* isopropanol, *filled triangle* ethyl acetate

The ln *x* versus (1/*T* − 1/*T*_mean_) curves of omeprazole sulfide in the eight solvents are shown in Fig. 3.

The standard molar Gibbs energy of solution $\Updelta {\text{G}}_{\text{soln}}^{0}$ can be calculated according to [34]:

9 $$ \Updelta G_{\text{soln}}^{\text{o}} = - RT_{\text{mean}} \times {\text{intercept}} $$

where the intercept used is that obtained in plots of ln*x* versus (1/*T* − 1/*T*_mean_). The standard molar entropy of solution is obtained from [30]:

10 $$ \Updelta S_{\text{soln}}^{\text{o}} = \frac{{\Updelta H_{\text{soln}}^{\text{o}} - \Updelta G_{\text{soln}}^{\text{o}} }}{{T_{\text{mean}} }} $$

The results of the standard Gibbs energy, enthalpy, and entropy of solution are shown in Table 4, together with *ξ*_*H*_ and *ξ*_*TS*_. The *ξ*_*H*_ and *ξ*_*TS*_ represent the comparison of the relative contributions to the standard Gibbs energy by enthalpy and entropy in the solution process, respectively [35].

11 $$ \% \xi_{H} = \frac{{\left| {\Updelta H_{\text{soln}}^{\text{o}} } \right|}}{{\left| {\Updelta H_{\text{soln}}^{\text{o}} } \right| + \left| {T\Updelta S_{\text{soln}}^{\text{o}} } \right|}} \times 100, $$

12 $$ \% \xi_{TS} = \frac{{\left| {T\Updelta S_{\text{soln}}^{\text{o}} } \right|}}{{\left| {\Updelta H_{\text{soln}}^{\text{o}} } \right| + \left| {T\Updelta S_{\text{soln}}^{\text{o}} } \right|}} \times 100. $$

**Table 4:** Thermodynamic functions relative to solution process of omeprazole sulfide in solvents at mean temperature

	$\Updelta H_{\text{soln}}^{\text{o}}$ (kJ·mol^−1^)	$\Updelta G_{\text{soln}}^{\text{o}}$ (kJ·mol^−1^)	$\Updelta S_{\text{soln}}^{\text{o}}$ (J·K^−1^·mol^−1^)	$\% \xi_{H}$	$\% \xi_{TS}$
Ethanol	43.401	7.483	119.770	54.717	45.283
95 % Ethanol	48.085	8.191	133.031	54.655	45.345
Ethyl acetate	34.744	11.876	76.255	60.307	39.693
Isopropanol	51.695	10.718	136.640	55.783	44.217
Methanol	49.457	7.315	140.528	53.993	46.007
Acetone	43.286	10.170	110.427	56.656	43.344
*n-*Butanol	43.956	8.357	118.708	55.252	44.748
*n-*Propanol	47.175	8.846	127.811	55.173	44.827

The conclusion can be drawn from Table 4 that the enthalpy and the standard Gibbs energy of solution of omeprazole sulfide are positive in the eight solvents, indicating the solution process of omeprazole sulfide in a total of eight solvents is endothermic. Moreover, the main contributor to the standard molar Gibbs energy of solution is the enthalpy during the dissolution, because the values of % *ξ*_*H*_ are ≥54 %.

## Conclusions

The solubility of omeprazole sulfide in a total of eight solvents has been measured from 280.35 to 319.65 K by a dependable experimental method and simple solubility apparatus. For all solvents, solubility is a function of temperature and increases with increasing temperature, but to each increment of temperature they responded with a definite change of solubility. The experimental data were fitted by using the modified Apelblat equation and *λh* equations and the Apelblat equation is more accurate than the *λh* equation for this system. The calculated solubility of omeprazole sulfide shows good agreement with the experimental values, and experimental solubility data from this work can be used for designing a purification process of omeprazole sulfide. The thermodynamic properties for the solution process including Gibbs energy, enthalpy, and entropy were obtained by the van’t Hoff analysis. The thermodynamic parameters values show that the solution process of omeprazole sulfide in a total of eight solvents is endothermic and the larger contributor to the standard molar Gibbs energy of solution is the enthalpy change during the dissolution.
